# Post-Mortem Diagnosis and Autopsy Findings in SARS-CoV-2 Infection: Forensic Case Series

**DOI:** 10.3390/diagnostics10121070

**Published:** 2020-12-10

**Authors:** Arthur-Atilla Keresztesi, Filip Perde, Andreea Ghita-Nanu, Carmen-Corina Radu, Mihai Negrea, Gabriela Keresztesi

**Affiliations:** 1 Ialomita County Institution of Forensic Medicine, 920013 Slobozia, Romania; arthurkeresztesi@gmail.com (A.-A.K.); andreea_nanu@yahoo.com (A.G.-N.); 2 Faculty of Medicine, “Carol Davila” University of Medicine and Pharmacy, 050455 Bucharest, Romania; filipvirgil@gmail.com; 3 Faculty of Medicine, “George Emil Palade” University of Medicine, Pharmacy, Science and Technology of Targu Mures, 540139 Targu Mures, Romania; 4 Department of Public Health, Faculty of Political, Administrative and Communication Science, “Babeș Bolyai” University, 400084 Cluj Napoca, Romania; 5 Department of Internal Medicine, Emergency Clinical County Hospital of Targu Mures, 540139 Targu Mures, Romania; gabriela.keresztesi@gmail.com

**Keywords:** SARS-CoV-2, COVID-19, autopsies, forensic, femur fracture

## Abstract

Towards the end of 2019, a novel coronavirus was identified as the culprit for a cluster of pneumonia cases in Wuhan, China. Since then, it has rapidly spread worldwide, affecting more than 43 million people, and in March 2020, the World Health Organization (WHO) declared it a pandemic. The purpose of the study is to present the findings of 15 forensic autopsies performed in Romania, on SARS-CoV-2 (severe acute respiratory syndrome coronavirus 2) positive subjects, and to present the case of one SARS-CoV-2 infected patient who experienced a violent death, as established during their autopsy. A total of 11 male and 4 female patients were autopsied, and SARS-CoV-2 infection was diagnosed post-mortem in two cases. The most frequent symptoms before death were dry cough, dyspnoea, and fever. Hypertension, ischemic cardiac disease, and a history of stroke were the most frequent associated diseases. The mean duration from the symptoms’ debut to a RT-PCR positive SARS-CoV-2 test was 3.7 days, while the mean survival time from the RT-PCR positive test was 4.2 days. A histological examination was performed in seven cases and revealed, in most of them, hyaline membranes, and mixed inflammatory cell infiltration of the interstitium, alveoli, and perivascular areas. In addition, all of the examined cases developed small vessel thrombosis. A case of violent death was also reported, regarding a 87-year-old male subject who suffered a femur fracture (domestic fall) and was diagnosed with SARS-CoV-2 infection the following day after surgery. After transfer to a COVID-19 (coronavirus disease-19) support hospital, during an episode of behavioral disorder, the patient jumped from the first floor window. Death occurred a few days later, and the cause was established as bronchopneumonia superimposed on SARS-CoV-2 infection. In conclusion, autopsies should be conducted while providing a safe environment for professionals to perform them, because they are crucial procedures that can help gain a better understanding of the role of SARS-CoV-2 infection in thanatogenesis.

## 1. Introduction

Towards the end of 2019, in Wuhan, China, a novel coronavirus was identified as the cause for a cluster of pneumonia, and since then, it has rapidly spread worldwide [1]. In February 2020, the World Health Organization (WHO) designated the disease as COVID-19 (coronavirus disease-19) and the novel coronavirus as SARS-CoV-2 (severe acute respiratory syndrome coronavirus 2). In March 2020, the WHO declared this outbreak a pandemic. Most infected individuals will experience mild-to-moderate respiratory illness, or even remain asymptomatic, while those with underlying comorbidities (cardiovascular disease, diabetes mellitus, chronic respiratory disease, kidney and liver disease, or cancer) are more likely to develop serious illness [2].

Since the emergence of the 2019 novel coronavirus infection in Wuhan, China, it has rapidly spread across the world, affecting more than 43 million people and causing over 1.1 million deaths worldwide, at the time this article was written [3]. Reported case counts are underestimated worldwide, as only a small amount of infections are diagnosed and reported. In the United States and in Europe, the rate of seropositivity (reflecting prior exposure to SARS-CoV-2) exceeds the rate of reported cases by approximately 10-fold or more [4,5]. In Romania, the number of positive cases exceeded 212,000 at the time this article was written, with more than 6400 deaths. In Ialomita County, one of the top five smallest counties in Romania, 934 cases were confirmed as of 27 August 2020, and 56 patients have died [3,6].

In patients with COVID-19 disease, the most common clinical symptoms are fever and cough, shortness of breath, and loss of smell and taste, in addition to other nonspecific symptoms, including headache, dyspnoea, fatigue, and muscle pain. Recent studies have shown that patients associating one or more of the following—diabetes mellitus, cardiovascular disease, chronic respiratory disease, cancer, or renal and hepatic dysfunction—are at higher risk for severe COVID-19 than children, who might be less likely to become infected or, if so, may develop milder symptoms or even remain asymptomatic [7,8].

In a report from the Chinese Center for Disease Control and Prevention, which included data from 44,672 confirmed cases of COVID-19 (positive viral nucleic acid test result on throat swab samples), 81% (36,160 cases) presented with mild symptoms, 14% (6168 cases) presented with severe disease (dyspnoea, hypoxia, or more than 50% lung involvement on imaging), and 5% (2087) of patients were critical (respiratory failure, shock, or multiorgan dysfunction) [9].

From the beginning of this pandemic, there has been a certain reluctance to perform autopsies, more so as the Robert Koch Institute had advised against it. The Royal College of Pathologists has issued an autopsy practice for SARS-CoV-2 infected cases, containing minimum requirements for performing safe autopsies [10,11].

The Romanian criminal procedure code states that in case of a violent or suspected to be violent death, a medico-legal (forensic) autopsy should be conducted. In these difficult times, as more and more doctors have been practicing defensive medicine as the state and medical law are not very well harmonized [12], secondary legislation regarding autopsies was issued after the beginning of the pandemic, with the purpose of protecting forensic teams, and not to expose them to unnecessary autopsies, within the limits of the law [13].

The purpose of the study is to present the findings after a series of forensic autopsies performed on SARS-CoV-2 positive subjects, and to present a case of violent death in a SARS-CoV-2 infected patient.

## 2. Materials and Methods

The present study is a post-mortem case study conducted between the 15 March 2020 and 27 August 2020 in the Forensic Institution of Ialomita County, Romania.

Consent was obtained from the Legal Guardian of the deceased, and the protocol was approved by the Ethics Committee of the Slobozia Emergency County Hospital (4/17.11.2020). From a total of 56 SARS-CoV-2 deaths, forensic autopsy was requested in 15 of them, according to the penal law—violent or suspected to be violent deaths, or deaths occurring in an institutionalized person.

External examination and complete autopsy was performed in all 15 subjects with COVID-19 infection, according to the official technique, by opening all luminal structures and lamellar incisions of all parenchymatous organs. The recommended protective measures were taken for the medical staff.

SARS-CoV-2 infection was confirmed from lung tissue samples or nasopharyngeal swabs using real-time PCR testing, which was supervised by the Romanian Public Health Department.

To conduct the study, demographic and clinical data were collected, such as age, sex, environment, pre-existing symptoms (fever, cough, chills, dyspnoea, fatigue, or stomach ache), and associated diseases (diabetes mellitus - DM, obesity, hypertension, history of stroke or myocardial infarction, neoplasia, ischemic cardiac disease, chronic alcohol consumption, or hepatitis B/C). For the classification of cases and clinical evolution, data were collected regarding the elapsed time from the symptoms’ onset to a SARS-CoV-2 positive test result, and survival time from the SARS-CoV-2 positive test.

Histological examination of the lung tissue was performed using samples from the perihilar region, fixed for a minimum of 72 h in a 10% formaldehyde solution with a neutral Ph. Hematoxylin–eosin (HE)-staining was used at a 200×-magnification. Immunohistochemical analyses for CD45RO (marker for T lymphocytes), CD20 (marker for B lymphocytes), CD68, and CK7 were performed in some cases.

The data collected were included in an electronic database using Microsoft Excel 2010 software (Microsoft Corporation, Bucharest, Romania). Statistical analysis was performed using Epi Info software (Centers for Disease Control and Prevention, Atlanta, GA, USA); Fisher’s exact test, Chi square Yates corrected Chi square, and T tests were used with a confidence level of 95%. A *p* value <0.05 was considered statistically significant. Continuous variables were reported as mean ± standard deviation (SD) and median, and categorical variables were the observed number of patients.

## 3. Results

During the five months analyzed, 934 SARS-CoV-2 infection cases were confirmed in the analyzed region; 56 deaths occurred, of which 15 (26.8%) were declared medico-legal cases. The mortality rate reported in the region was 6% in the mentioned period.

From the 15 cases, three of them were sudden deaths that occurred at home, three of them were deaths in the emergency room (ER) of different hospitals in the county, and nine of them were in-hospital deaths (seven occurred in previously institutionalized subjects). In two cases of sudden death, SARS-CoV-2 infection was diagnosed post-mortem, using lung tissue samples or nasopharyngeal swabs collected during autopsy.

The gender and environment background of the 15 cases revealed 4 female and 11 male subjects, while 10 came from an urban environment and 5 from a rural one.

In Table 1, all of the group characteristics are presented for each subject.

The mean age of the 15 cases was 68 ± 20 years and the median was 71 years, ranging from 24 years to a maximum of 96 years.

**Figure 1:**
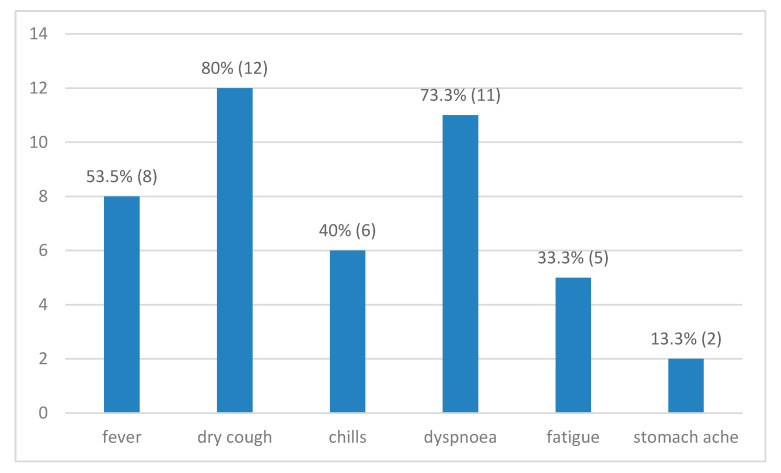
Associated symptoms.

The presence of a certain symptom, the associated diseases, and the mean age did not associate or differ significantly (*p* > 0.05) with gender (male subjects were 66 ± 22 years old, while female subjects were older, 76 ± 16 years—Figure 1).

**Figure 2:**
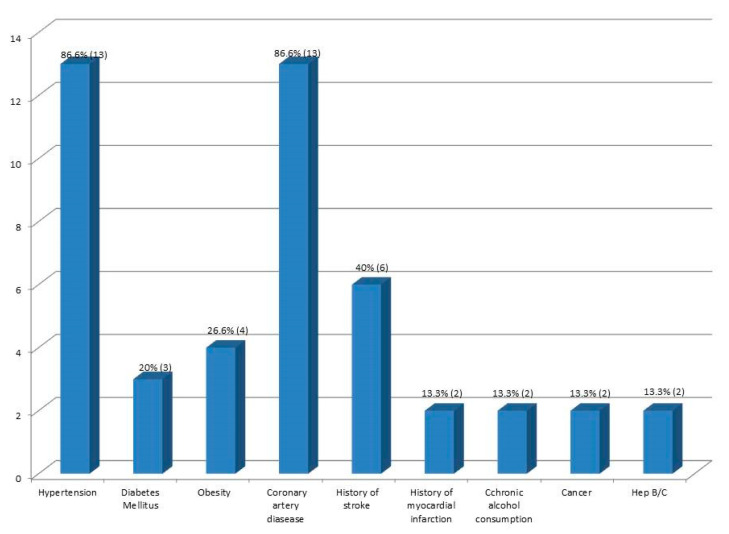
Associated diseases.

Most patients presented hypertension, coronary artery disease, and a history of stroke (Figure 2). The median number of comorbidities in these cases was three, and ranged from no comorbidity (in one case under the age of 30 years) to a maximum of six.

The mean period of time elapsed from the symptoms’ debut to a SARS-CoV-2 positive test was 3.7 ± 1.7 days, with a minimum of 6 h (according to family members) to a maximum of 7 days. There was no statistically significant difference (*p* > 0.05) between rural and urban areas.

Regarding the mean period of survival time from SARS-CoV-2 infection diagnosis (positive test result) to death, the result was 4.2 ± 4.5 days, ranging from no survival time (in two cases diagnosed post-mortem) to a maximum of 12 days. The statistical analysis yielded a significant difference regarding gender—female subjects survived a mean 1.2 ± 1.2 days, while male subjects survived a mean period of 5.2 ± 4.8 days (*p* = 0.02).

The time from the onset of symptoms to death ranged from 6 h to 17 days, with a mean and median of 9 days; male patients survived longer (9 days) than female patients (5 days), with a statistically significant difference (*p* < 0.05).

Macroscopic examination of the lungs revealed congested, firm, heavy lungs with areas of edematous tissue and patchy involvement, as well as areas of diffuse consolidation. In most cases, the superior airways were mucus free.

**Figure 3:**
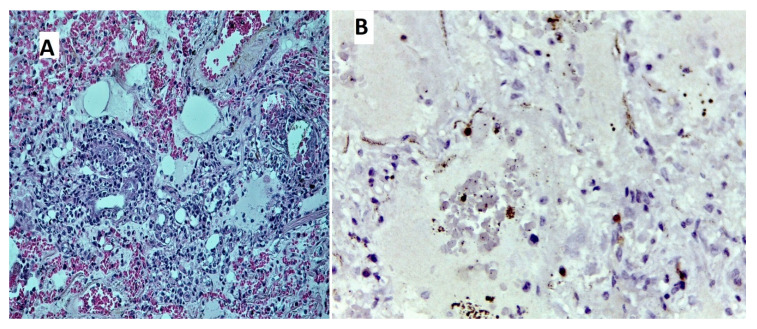
Tissue sample from left lung parenchyma, from the perihilar region, fixed in a 10% formaldehyde solution with a neutral Ph for a minimum of 72 h. (**A**) Diagnosis: hematoxylin–eosin (HE)-staining (200×-magnification). Small vessels with early organizing thrombus with red blood cells and surrounding fibrin accumulations (fibrino-hematic), leukostasis, and congestion. Alveolar walls are thickened due to acute distention of capillaries and interstitial oedema. Alveolar lumen is filled with pale-eosinophilic, finely granular content (transudate)—*alveolar oedema*—and neutrophilic and macrophagic elements. (**B**) Immunohistochemistry (400×-magnification), CD45RO positive, mostly in the interstitium.

Histological examination was performed in 7 of the 15 cases. In four cases, microscopic evaluation of the lung tissue revealed areas of bronchopneumonia due to superimposed bacterial infection; as a landmark element, diffuse alveolar damage in the florid phase with exudative lesions, associating hyaline membranes, and oedema was noticed in five cases, with mixed inflammatory cell infiltration, including lymphocytes and plasmocytes in the interstitium, and perivascular areas, fibrin, and leukocytes in the alveolar and bronchiolar areas. Microthrombi in small vessels and capillaries were observed in all cases (Figure 3). Examination of the kidney tissue samples revealed, in all cases, acute epithelial tubular necrosis.

Immunohistochemistry was performed in four cases. The CD20 was negative in all of the cases, CD45RO was variably positive, with a moderate reaction in T lymphocytes and CD68 moderately expressed in macrophages. CK7 was rarely observed in some intra-alveolar epithelia.

From the 15 autopsied cases, in 14 cases, the manner of the death was considered to be natural, and in one case the death was ruled violent. This one case is presented in the current manuscript.

The direct cause of death in 13 cases was SARS-CoV-2 massive bilateral pneumonia, in one case pulmonary carcinoma with extensive necrosis, and in the last case, bronchopneumonia superimposed on SARS-CoV-2 pneumonia, following a femur fracture.

### Case Presentation

**Figure 4:**
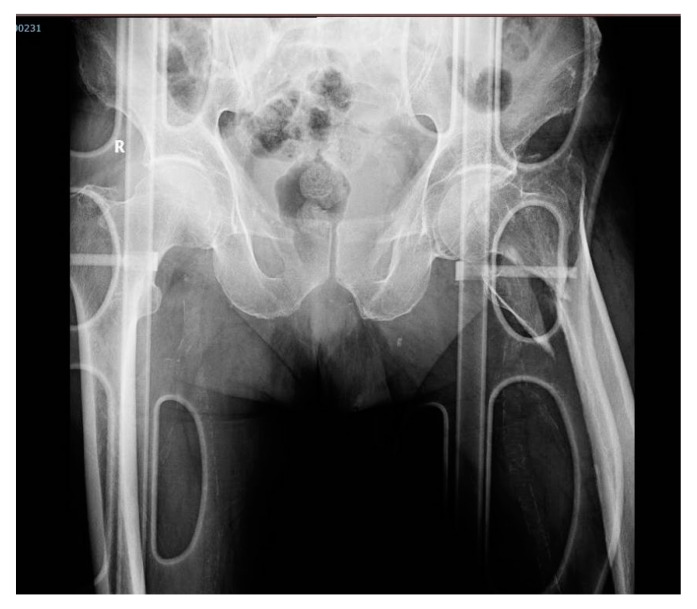
Pelvic X-ray upon arrival, in anteroposterior incidence. Description: Male subject on gurney. Right hip with minimal narrowing of the joint space. Left hip showing a left comminuted femur fracture in the upper ⅓ segment, with displaced bone fragments and minimum angulation.

An underweight 87-year-old male subject with a history of hypertension and dementia, presented an accidental fall at home and was rushed to the local emergency county hospital on the same day. Upon admission, he presented 147/75 mmHg blood pressure, 60 bpm heart rate, SpO2 98%, and a temperature of 36.9 °C. A hip X-ray was performed, revealing a left pertrochanteric femur fracture (Figure 4). In addition, a nasopharyngeal swab specimen for SARS-CoV-2 was collected.

**Figure 5:**
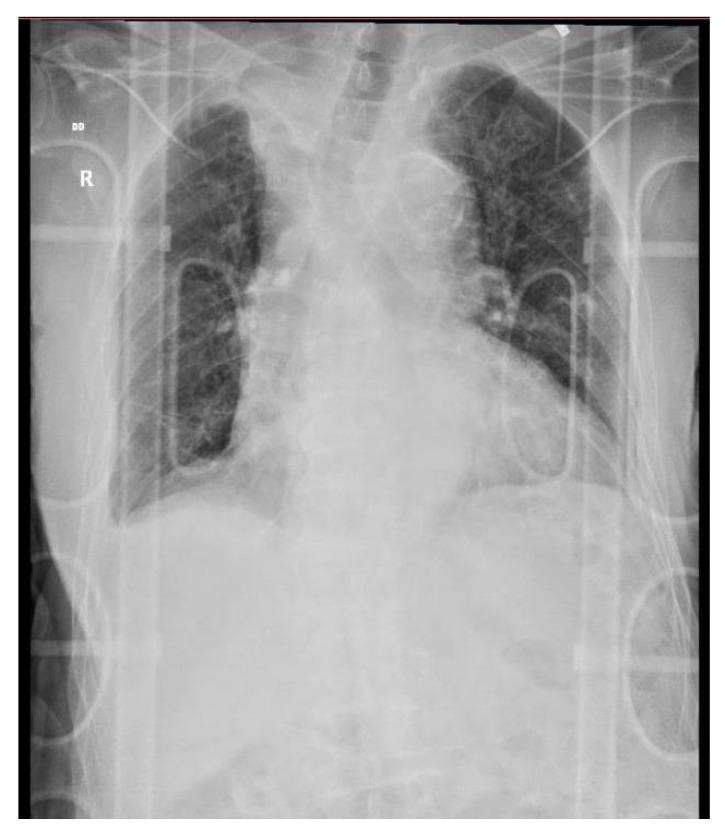
Chest X-ray upon arrival as per pre-surgery protocol, anteroposterior incidence. Description: diffuse, bilateral, reticular pattern. Possible right upper lobe atelectasis. Increased area of projection of the heart and mediastinum. Minimal right pleural effusion. No signs of rib fractures.

**Figure 6:**
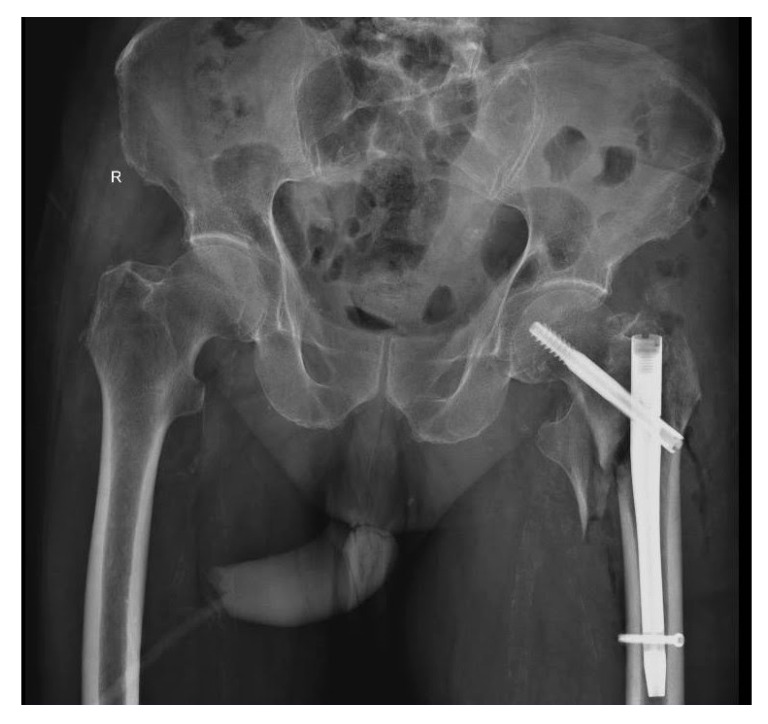
Post-surgery pelvic X-ray, anteroposterior incidence. Description: male subject, urinary catheter. Right hip with minimal narrowing of the joint space. Left hip with osteosynthesis material (intramedullary rod) in the proximal segment of the femur with distal nailing. Bone fragment with dorsal and internal displacement. Coxo-femural spaces maintained. Acetabular surfaces with sclerosis.

The next day, before surgery, the patient underwent a chest X-ray (Figure 5) and blood work (leucocyte 10.93 × 10^3^/uL; haemoglobin 8 g/dL; Htc 24%; serum creatinine 2.07 mg/dL; urea 100 mg/dL). A left femur intramedullary nail was placed (Figure 6), and one unit of blood was transfused.

The SARS-CoV-2 results came back positive the following day and the patient was transferred to a COVID-19 support hospital with the following diagnosis: “Left pertrochanteric fracture. Hypertension. Posthemorrhagic anemia”.

Upon arrival at the hospital, the patient presented with SpO2 87% and 140/90 mmHg blood pressure. A new chest X-ray was performed, which yielded bilateral perihilar and basal lung consolidations, with micronodular opacities. Shortly after admission, the patient presented a major episode of behavioral disorder with physical and verbal aggression towards the medical professionals. The medical team caring for the patient decided to move him to an isolated ward, in order to be supervised more carefully. During transfer, despite the recent surgery, the patient managed to jump from the first floor window (a height of approximately 3 m). Emergency medical care was given, the patient remaining conscious and stable. He was transferred back to the local emergency county hospital with the following diagnosis: “COVID-19 confirmed interstitial pneumonia. Politrauma by falling from another level. Acute respiratory failure”.

**Figure 7:**
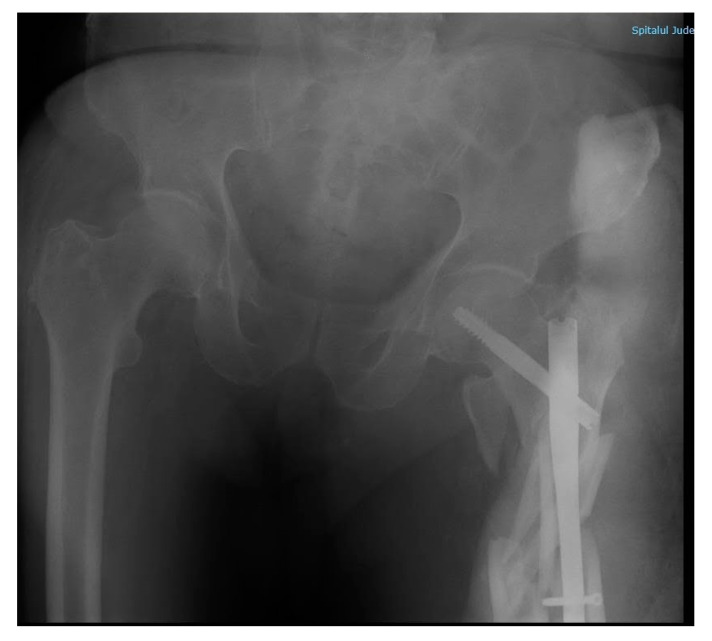
Hip X-ray after 3-m height fall, anteroposterior incidence. Description: male subject. Right hip with minimally narrowed joint space. Left hip in the ⅓ proximal of the femur with osteosynthesis material (femoral rod distally nailed) slightly moved, degraded. Comminuted fracture with bone fragments misaligned in the proximal diaphysis of the left femur, with angulation of fragments. Loss of contour between normally continuous line from medial edge of femoral neck and inferior edge of the superior pubic ramus. Minimally impacted left femoral neck.

Upon arrival, the patient presented with a 92/55 mmHg blood pressure and SpO2 92%; the surgical examination revealed no cranial, thoracic, or abdominal bleeding. Blood work panel (leucocytes 14.45 × 103/uL; haemoglobin 9.8 g/dL; Htc 29.5%; serum creatinine 1.73 mg/dL; urea 83 mg/dL; TGO 169.3 U/L; TGP 194 U/L) and a left lower limb X-ray were repeated (Figure 7). The X-ray revealed a left femur comminuted fracture with osteosynthesis material destruction. The patient was sedated with propofol and fentanyl, in order to avoid other incidents.

On day six since the first admission, the patient spiked a fever (39.2 °C), with a SpO2 of 97% on the oxygen mask, and shortly after midnight, his neurological state significantly deteriorated. He became hemodynamically and respiratory unstable (84/48 mmHg, SpO2 62%) and suffered a cardiac arrest, unresponsive to CPR maneuvers. As the death was ruled as suspected to be violent (potentially a complication of the femur fracture), a forensic autopsy was requested.

**Figure 8:**
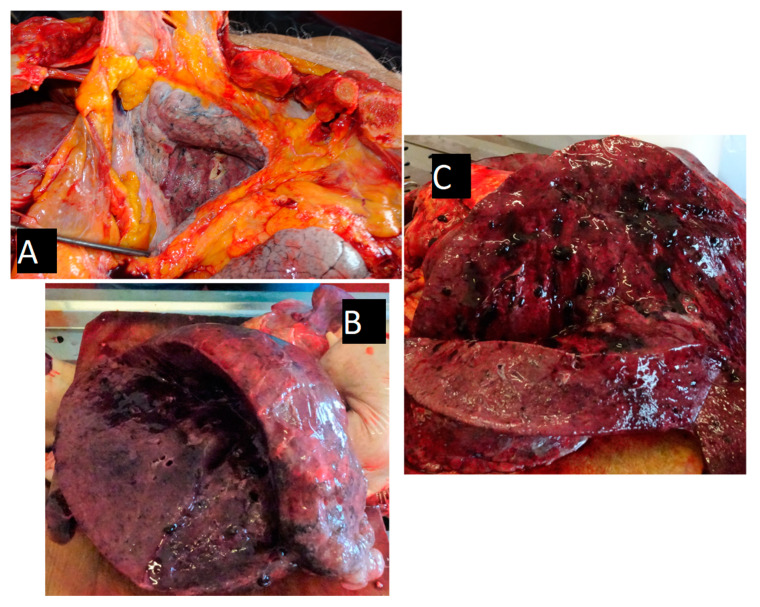
Macroscopic lung aspect during autopsy. Description: Both the left (weight = 740 g, normal = 110–650 g) and the right lungs (weight = 850 g, normal = 150–700 g) were enlarged. No accumulation of fluids were detected in the pleural cavities or pericardial cavity. (**A**) The right pleural cavity presented massive pleural adhesions, with thickened, whitish pleura. (**B**) The left lung showed minimum hemorrhagic petechiae on the pleural surface without signs of pleurisy. A cross section of the lung revealed pulmonary edema and a diffusely firm, condensed, with loss of crepitations in the parenchyma. All of the lobes were affected equally. The bronchi presented little yellowish mucus and the vasculature was patent. (**C**) The parenchyma had pink areas ranging to dark red or brown areas in color with scattered ill-defined hemorrhagic areas; in the right lung parenchyma, mucus plugging was present. The hilar lymph nodes were slightly enlarged, and were black on the cut surface.

**Figure 9:**
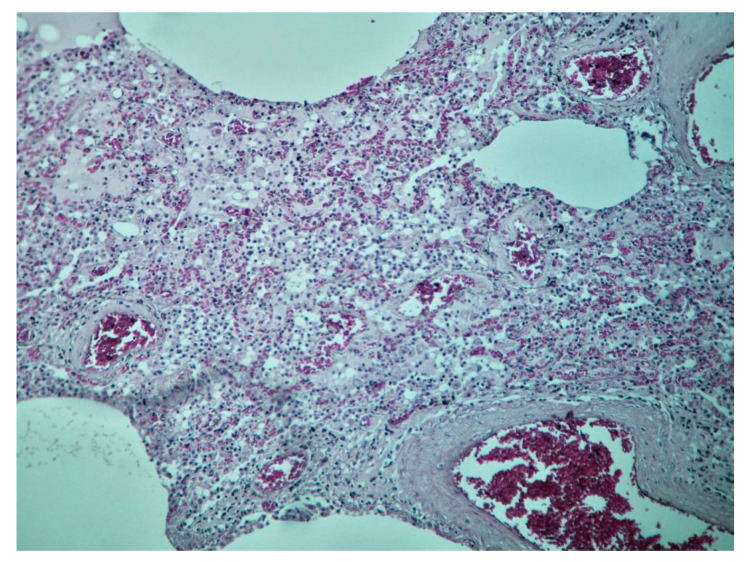
Tissue sample from the left lung parenchyma, from the perihilar region, fixed in a 10% formaldehyde solution with neutral Ph for a minimum of 72 h. Diagnosis: hematoxylin–eosin (HE)-staining (200× magnification), lung parenchyma showing alveolar spaces filled with a pale-eosinophilic, finely granular content (transudate; alveolar oedema), neutrophils and scattered macrophages, and vascular congestion within alveolar walls (mixed inflammatory cell infiltration in the alveoli).

**Figure 10:**
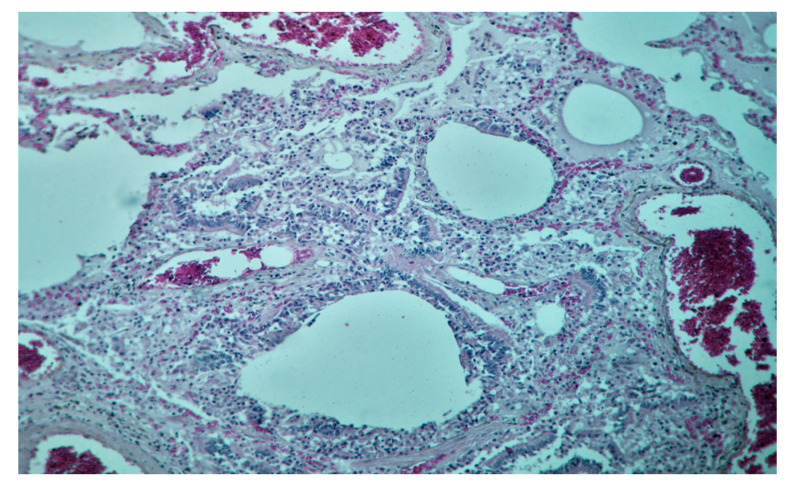
Tissue sample of the left lung parenchyma, from the perihilar region, fixed in a 10% formaldehyde solution, with neutral Ph for a minimum of 72 h. Diagnosis: hematoxylin–eosin (HE)-staining (200×-magnification), bronchiolar lumens with damaged epithelium, neutrophils, and few macrophages. Surrounding alveolar spaces with pale-eosinophilic, finely granular content (*oedema*)*,* and neutrophilic exudate.

Autopsy findings: External examination of the body showed bruises and lacerations on the head, thorax, and abdomen, with a surgical wound and abnormal mobility in the upper third portion of the left thigh. The deceased was 166 cm tall and was underweight. The internal examination revealed leptomeningeal oedema, massive right pleural adhesions, and purple pale lungs with an increased consistency, with dark blood stagnation and reduced yellowish mucus on sections (Figure 8). Other findings included diffuse myocardial scarring, advanced coronary and aortic atherosclerosis, spleen fibrosis, nephrosclerosis, and an operated pertrochanteric left femur fracture, with destruction of the osteosynthesis material. Histopathological examination yielded moderate alveolar oedema, mixed inflammatory cell infiltration in the peribronchiolar and alveolar areas (including fibrin and leukocytes), and perivascular and bronchiolar lymphoplasmacytic infiltrate (Figure 9 and Figure 10). The manner of death was ruled as violent. The underlying cause of death was bronchopneumonia (bacterial infection) superimposed on SARS-CoV-2 pneumonia (RT-PCR confirmed), developed in the days following a left femur fracture, which was surgically treated and subsequently refractured. An indirect causal link between the femur fracture and death was established. 

## 4. Discussion

Autopsies are considered the gold-standard for establishing the cause of death, preexisting associated diseases, and the implication of SARS-CoV-2 infection in the cause of death.

As reported in numerous studies [14,15,16], advanced age, along with the presence of comorbidities, increases the risk of developing serious or fatal COVID-19 infection.

We reported a detailed analysis of 15 autopsied COVID-19 cases, including associated diseases and symptoms of COVID-19. The patients in our study reached a median age of 71 years, a similar result to those presented by Elezkurtaj et al. [16] in their study on 26 cases (mean age of 70 years).

Dry cough, dyspnoea, and fever were present in more than half of the cases, with a mean of 3.7 days elapsed from the symptoms’ debut to SARS-CoV-2 infection confirmation, results similar to other studies [17,18]. Elsoukkary et al. [19], in their study on 32 COVID-19 autopsies, reported similar values regarding the presence of shortness of breath, cough, and fever, in this order.

The median survival time from symptoms’ onset to death was 9 days in our study, and ranged from 6 hours to a maximum of 17 days, while Elezkurtaj et al. [16] reported higher survival intervals, with a median of 26 days and a maximum of 51 days. The data in our study yielded that female patients survived a significantly shorter period of time compared with men, but male patients were infected more often—with broadly similar patterns being reported by other studies [20].

Histological examination was performed only in seven cases—three sudden deaths, three ER deaths, and one in-hospital death (suspected to be violent death). The rest of the eight cases were in-hospital deaths, with repeated positive RT-PCR SARS-CoV-2 tests and well documented COVID-19 disease.

Autopsy reports of the lungs in COVID-19 cases have mainly revealed diffuse alveolar damage (DAD) or acute lung injury [21,22]. A combination of hyaline membranes in the alveoli, mixed inflammatory cells in the interstitium, alveoli and perivascular areas, and small vessel thrombosis were consistent findings in our cases, as frequently reported in other studies [23]. The same authors also reported a 60% incidence of other lung co-infections (mainly bacterial), tumors, or aspiration pneumonia, also consistent with our findings.

In an extensive literature review conducted by Maiese et al. [24], which included 28 scientific papers with a total of 341 COVID-19 autopsy cases, the most prominent histological feature in the lungs was diffuse alveolar damage with hyaline membranes formation and small pulmonary vessel thrombosis, consistent with the findings presented in this paper.

Acute tubular necrosis in the kidneys was a constant finding in our study, and in other studies as well [19]. Electron microscopy studies reported the presence of viral particles within both the tubular epithelium and podocytes, while RT-PCR on the kidney tissue obtained from biopsy, urine, and serum were negative for SARS-CoV-2 [25].

The prothrombotic state induced by COVID-19, well documented in numerous studies [21,23], and also histologically in seven of our cases, raises the need for an improvement in management strategies. An anticoagulation algorithm is proposed based on disease severity [26], but it is highly recommended to also assess the bleeding risk in these patients, considering that severe forms of COVID-19 affect mostly older subjects and the risk of bleeding increases with age [27]. Maiese et al. [28] raise the problem of viral sepsis with the release of pro-inflammatory cytokines, with a role in interstitial pneumonia aggravation and hypercoagulability, and propose early prophylactic immunomodulatory and anticoagulant therapy.

The national SARS-CoV-2 continuously updated guideline, at the time of this study, stipulated that a patient is considered to be healed and safe for discharge if he is free of symptoms and has two consecutive negative RT-PCR tests, performed in a minimum of a 24-hours interval.

Another four autopsied cases, which were not included in the present study, involved COVID-19 patients with two negative RT-PCR tests at the time of discharge from the hospital, that died at home in the following week, thus raising the suspicion of violent deaths, and autopsies were requested. The autopsies of all four cases yielded massive bilateral pneumonia, which was considered to be the cause of death. Although the patients were free of the SARS-CoV-2 virus, according to the negative RT-PCR test, the lung damage was already present, and more so in an extensive way.

We also reported the case of an elderly patient, having survived a femur fracture, initially properly repaired surgically, SARS-Cov-2 infection, a subsequent fall from a 3-m height with refractured femur and osteosynthesis material degradation, and dying of bronchopneumonia (bacterial infection) histologically confirmed, superimposed on SARS-CoV-2 infection and lung damage. The manner of death was ruled as violent, indirectly linked to the femur fractures.

Thakrar et al. reported an increase in 30-day mortality among patients with hip fractures during the first 30 days from the outbreak of COVID-19 pandemic, a 2.4-fold increase in mortality being associated with SARS-CoV-2 infection [29].

We consider a limitation of our study to be the small sample size. Furthermore, the subjects included in the current study had the median age of 71 years, which is similar to the reported age distributions of inpatient non-survivors in Wuhan [14], but is lower than that suggested by other epidemiologic data from Italy on SARS-CoV-2 infected patients [30]. The interpretation of autopsy results and conclusions on the health impact of COVID-19 requires careful consideration of the study population.

## 5. Conclusions

The presence of comorbidities worsens the outcome of SARS-CoV-2 infection, frequently leading to death, especially in older patients. Hypertension, ischemic cardiac disease, and a history of stroke were the most frequent associated diseases in our study.

In the days leading to a RT-PCR SARS-CoV-2 test, the majority of patients presented dry cough and dyspnoea, with a mean period of 3.7 days from the symptoms’ debut to COVID-19 diagnosis confirmation, and a mean time of 9 days from symptoms’ onset to death. SARS-CoV-2 infection was diagnosed post-mortem in two cases.

Macroscopic autopsy findings yielded a typical aspect suggestive of SARS-CoV-2 affected lungs, these being firm, condensed, heavy, with a loss of crepitations. Histological examination displayed variable characteristics, with hyaline membranes and small vessel thrombosis being reported most frequently. In addition, superimposed bacterial infection was a common finding in our cases.

In one conducted autopsy, the manner of death was ruled as violent and with an indirect causal link to the traumatic event leading to death.

In conclusion, autopsies should be conducted while providing a safe environment for professionals to perform them, because they are crucial procedures that can help gain a better understanding of the role of SARS-CoV-2 infection in thanatogenesis.

## Figures and Tables

**Table 1:** Group characteristics.

Case No.	Gender	Age (y)	Hypertension	DM	Obesity	CAD	History of MI	History of Stroke	Chronic Alcohol Consumption	Cancer	Hep B/C Virus	Survival after RT-PCR Test (days)
1	M	60	yes	yes	yes	yes	no	no	no	no	yes	0
2	M	77	yes	no	no	yes	yes	yes	yes	yes	no	0
3	F	58	yes	no	yes	yes	no	no	no	no	no	0
4	M	87	yes	no	no	yes	no	no	no	no	no	7
5	F	70	yes	no	yes	yes	no	no	no	no	yes	1
6	M	87	yes	no	no	yes	no	no	no	no	no	7
7	M	92	yes	no	no	yes	no	no	no	no	no	2
8	M	71	yes	yes	no	yes	no	yes	yes	yes	no	9
9	F	81	yes	yes	no	yes	no	yes	no	no	no	3
10	M	29	no	no	yes	no	no	no	no	no	no	0
11	M	63	yes	no	no	yes	yes	no	no	no	no	1
12	M	24	no	no	no	no	no	no	no	no	no	9
13	M	71	yes	no	no	yes	no	yes	no	no	no	12
14	F	96	yes	no	no	yes	no	yes	no	no	no	1
15	M	68	yes	no	no	yes	no	yes	no	no	no	12

DM—diabetes mellitus; CAD—coronary artery disease; MI—myocardial infarction.
